# Circadian rhythm disruption is associated with an increased risk of sarcopenia: a nationwide population-based study in Korea

**DOI:** 10.1038/s41598-019-48161-w

**Published:** 2019-08-19

**Authors:** Youn I Choi, Dong Kyun Park, Jun-Won Chung, Kyoung Oh Kim, Kwang An Kwon, Yoon Jae Kim

**Affiliations:** Department of Gastroenterology, Gil Medical Center, Gachon University, Incheon, Korea

**Keywords:** Geriatrics, Risk factors

## Abstract

Reduced sleep quality is associated with an increased risk of sarcopenia. However, the potential effects of disturbing the regular circadian rhythm, as occurs with shift work, on the risk of sarcopenia have not been established. Data from 9105 workers from the 2008–2011 Korean National Health and Nutrition Examination Survey were analyzed. Sarcopenia, measured by dual-energy X-ray absorptiometry, was defined as one standard deviation below the mean of the appendicular skeletal muscle/body mass index value of a young reference group. Compared to the group that had never experienced shift work, the odds ratio (OR) for sarcopenia with a 95% confidence interval (95% CI) for the shift work group was 1.7 (1.5–1.9); the association remained even after adjusting for confounding variables, including age, sex, total fat mass, insulin resistance profile, smoking, alcohol intake, diet, and physical activity. The results of the subgroup analysis indicated that the highest risk of sarcopenia was among workers engaging in shift work with an irregular schedule (OR 1.8, 95% CI 1.3–2.4). Disruption of circadian rhythm by shift work was associated with increased risk of sarcopenia. Intervention strategies are needed to prevent sarcopenia in shift workers.

## Introduction

Sarcopenia is defined as the progressive, generalized loss of skeletal muscle mass, strength, and function^1,2^. Sarcopenia is divided into primary and secondary sarcopenia^2^. Primary sarcopenia is regarded as a normal part of the aging process with no other cause^2^, whereas secondary sarcopenia is related to conditions other than aging, such as chronic inflammatory disease^2^. Although sarcopenia is primarily a disease of the elderly, it is also seen in younger individuals.

Sarcopenia is associated with multiple adverse outcomes, including various diseases such as cardiovascular disease^3,4^, stroke^5^, liver disease^6–8^, pulmonary disease^9^, chronic kidney disease^10,11^, gastrointestinal disorders^12^, malignancy^13^, postoperative morbidity^14,15^, emotional disorders^16^, and all-cause mortality^17^. Therefore, it is essential to determine the prevalence and causes of sarcopenia to improve patients’ outcomes.

There are a number of known causes of sarcopenia, including aging, disuse, malnutrition, and chronic inflammatory diseases, such as osteoporosis. Several recent studies have indicated that decreased sleep quality and long or short sleep duration may also play roles in the development of sarcopenia^18–20^.

However, whether circadian rhythm disturbance is associated with sarcopenia has not been examined, even though maintaining circadian rhythm is known to be important for sleep quality. Here, we examined whether shift work^21^, which is known to disturb circadian rhythm, was associated with an increased risk of sarcopenia using data from a nationally representative cohort study in Korea: the Korean National Health and Nutrition Examination Survey (KNHANES).

## Method

### Institutional ethic review board approval of the study design

The institutional review board of Gil Medical Center reviewed the study protocol (IRB approval no: GC IRB 2018705) and approved the research. All research was performed in accordance with national guidelines and regulations. In this study, we used the data from the KNHANES which is a national surveillance system that has been assessing the health and nutritional status of Koreans since 1998, and its data is opened to public. The need for written informed consent was waived by the Ethics Committee.

#### Study population

The study was based on the 5th Korean National Health and Nutrition Examination Survey (KNHANES) conducted in 2008–2011 by the Korea Centers for Disease Control and Prevention, which aimed to estimate the health status of the Korean population^22^. All study participants provided informed consent. KNHANES enrolled representative subjects selected by stratified multistage sampling^22^. The variables in KNAHENS regarding socioeconomic factors, health status, and lifestyle factors including smoking, alcohol ingestion, physical activity, and dietary patterns were collected from self-administered questionnaires^22^.

Among 37,753 participants, subjects < 19 years old (n = 8835) and those with missing data (n = 541) were excluded from the present study. Therefore, a total study population consisting of 9,105 workers of all types was included in the final analysis.

#### Definition of sarcopenia

We defined sarcopenia in three different ways in accordance with the consensus report of the Asian Working Group for Sarcopenia, as below^1,23^: (1) weight-adjusted percentage muscle mass (ASM/weight squared) below the lowest quintile computed for the study population; (2) height-adjusted percentage muscle mass (ASM/height squared) below the lowest quintile computed for the study population; (3) body mass index-adjusted percentage muscle mass (ASM/body mass index) below the lowest quintile computed for the study population in

Body fat (kg) and appendicular skeletal muscle mass (ASM, kg) were measured through dual-energy X-ray absorptiometry (DISCOVERY-W fan-beam densitometer; Hologic, Inc., Bedford, MA)^22^. Among the total study population, subjects below the median value and subjects above the median value of the total study group were designated as the low and high total body fat mass groups, respectively. In the same manner, the low and high axial skeletal muscle mass groups were defined as subjects below the median and above the median axial skeletal muscle mass, respectively.

#### Definition of type of work and shift work

Type of work was categorized according to occupation: blue-collar workers (simple labor, agriculture, forestry, and fisheries), white-collar workers (professionals and office workers), and pink-collar workers (service workers)^22^.

Workers were divided into those who worked only in the daytime (regular daytime workers) and those with other work schedules (alternative shifts).

#### Other variables

Dietary patterns were assessed via single 24-h recall^19,22^. Individual energy and nutrient intakes were calculated using the seventh Food Composition Table prepared by the Korean National Rural Resources Development Institute^19,22^. Trained interviewers instructed respondents to recall and describe all foods and beverages consumed during the previous day^19^. Values indicating “excessive,” “appropriate,” and “lacking” in terms of nutrient status were those of the Dietary Reference Intakes for Koreans, revised in 2015. Exercise patterns were classified in accordance with exercise type and frequencies per week, such as walking exercise frequency and strength exercise frequency^19,22^. Smoking status was divided into nonsmoker and current smoker based on the response to the question “Do you currently smoke?^19,22^.” Alcohol drinking status was assigned based on the response to the question “Did you drink more than one glass of liquor during the last month?^22^.” Sleep duration (h/d) was self-reported as an answer to the question “How long do you usually sleep at night?”^22^. Household income was calculated as the sum of the monthly income of all family members and classified into groups including lowest (first quantile), middle-low (second quantile), middle-high (third quantile), and highest (4^th^ quantile)^22^. Education status was divided into four classes; education for 6 years or less as elementary school; 7–9 years as middle school; 10–12 years as high school; and 13 years or over as college or university. Homeostatic model assessment for insulin resistance (HOMA-IR) was defined as below; HOMA-IR = [Fasting blood insulin (µU/mL) × [Fasting blood glucose (mmol/L)]/22.5.

#### Statistical analysis

Continuous and categorical variables are expressed as the mean ± standard deviation (SD) and number with percentage (±SE), respectively. General characteristics were presented according to 1) the experience of shift work among the total population, and 2) the presence of sarcopenia among the shift work group. To compare 1) the shift work group vs the non- shift work group, and 2) members of the shift work group with sarcopenia vs members of the shift work group without sarcopenia, t-test and the chi-squared test were used. Odds ratios (ORs) and 95% confidence intervals (CIs) for the associations between shift work experience and sarcopenic status were analyzed using multivariate logistic regression analyses with or without adjustment for confounding factors. Statistical analyses were performed using SPSS ver. 20 for Windows. All tests were two-sided, and *P* < 0.05 was taken to indicate statistical significance.

## Results

### Characteristics of the study population according to shift work

A total of 9105 workers were included in our analyses. There were no significant differences in education level, income status, presence of a spouse, or medical history between non-shift workers and shift workers (Table 1). Hemoglobin A1C level (HbA1C), high-density lipoprotein (HDL), BMI, and axial skeletal muscle mass index were significantly different between the two groups, whereas fat mass index and waist circumference were not significantly different between the two groups (Table 1).

**Table 1:** General Characteristics of study population according to experience of shift work.

	Shift work− (N = 8,053)	Shift work+ (N = 1,052)	*P*
**Age, mean age (SD)**	46.2 ± 14.9	45.5 ± 14.4	0.2
**Male, n(%_)**	4,079 (50.7%)	715 (68.0%)	<0.001
Work type			<0.001
White/pink/blue color	3,510 (43.8%)/1,549 (19.3%)/2,957 (36.9%)	264 (25.3%)/244 (23.3%)/538 (51.4%)	
Education			0.6
<Elementary/middle/high/≥college	1,594 (19.8%)/873(10.3%)/2,809 (34.9%)/2,773 (34.5%)	157 (14.9%)/130 (12.4%)/511 (48.6%)/254 (24.1%)	
Household income(quantile)			0.2
1^st^/2^nd^/3^rd^/4^th^(highest)	1,860 (23.3%)/2,056 (25.8%)/2,090 (26.2%)/1,974 (24.7%)	205 (19.8%)/298 (28.8%)/273 (26.4%)/260 (25.1%)	
**Existence of spouse**	6,566(81.5%)	856 (81.4%)	0.7
Medical history			
Hypertension	1,355 (16.8%)	194 (18.4%)	0.4
Hyperlipidemia	619 (7.7%)	89 (8.5%)	0.5
Diabetes	491 (6.1%)	61 (5.8%)	0.8
Stroke	85 (1.1%)	11 (1.0%)	0.9
Cardiovascular disease	138 (1.7%)	19 (1.8%)	0.9
Depression	254 (3.2%)	22 (2.1%)	0.5
Laboratory data			
HbA1C, %	5.8 ± 1.0	6.1 ± 1.4	<0.001
Total cholesterol, mg/dL	188.8 ± 35.9	187.2 ± 35.4	0.2
HDL, mg/dL	49.7 ± 12.0	48.5 ± 11.6	0.004
BMI, kg/m2			0.002
<18.5	382 (4.9%)	374 (36.6%)	
18.5–22.9	3,131 (40.2%)	374 (36.6%)	
23.0–24.9	1,751 (3.7%)	246 (24.1%)	
25.0–29.9	2,230 (28.7%)	315 (30.9%)	
30.0−	288 (3.7%)	49 (4.8%)	
Waist circumference (cm)			0.2
low	6,062 (75.7%)	769 (73.9%)	
high	1,945 (24.3%)	272 (26.1%)	
Total body Fat mass group			0.8
low	5,239 (65.1%)	689 (65.5%)	
high	2,814 (34.9%)	363 (34.5%)	
Axial skeletal Muscle mass group			<0.001
low	2,874 (35.7%)	503 (47.8%)	
high	5,179 (64.3%)	549 (52.2%)	
Fat muscle mass index			<0.001
highMlowF	3,176 (39.4%)	322 (30.6%)	
lowMlowF	2,063 (25.6%)	367 (34.9%)	
highMhighF	2,003 (24.9%)	227 (21.6%)	
lowMhighF	811 (10.1%)	136 (12.9%)	

Abbreviations: HDL, high density lipoprotein; BMI, body mass index; ASM, axial skeletal muscle; LowMhighF, low muscle high fat group; HihgMlowF, high muscle low fat group; HighMhighF, high muscle high fat; HighMlowF, high muscle low fat.

### Baseline health behaviors according to shift work type

Health behaviors, including diet, physical activity, smoking, alcohol ingestion, and sleep duration were examined according to the type of work schedule (Table 2). Energy excess, current smoking, current alcohol ingestion, frequency of strength exercise, and sleep duration of less than 5 hours per night were more prevalent in the shift worker group (Table 2). However, carbohydrate intake, protein intake, fat intake, and frequency of walking exercise were not significantly different between shift work and non-shift work groups (Table 2).

**Table 2:** Health behaviors of study population according to experience of shift work.

	Shift work− (N = 8,053)	Shift work+ (N = 1052)	*P*
Energy (kcal/day)			0.04
Excess	3,798 (55.4%)	529 (59.0%)	
Carbohydrate (g/day)			0.9
>65% of total energy	5,278 (65.5%)	692 (65.8%)	
Protein (g/day)			0.9
≥20%of total energy	1,857 (23.1%)	241 (22.9%)	
Fat (g/day)			0.2
>30% of total energy	1,831 (22.7%)	222 (21.1%)	
Smoking status			<0.001
Never-smoking	4,205 (52.2%)	413 (39.3%)	
Abstinence (ex-smoker)	1,622 (20.1%)	176 (16.7%)	
Current smoker	2,226 (27.6%)	463 (44.0%)	
Alcohol consumption frequency in past 1 year			<0.001
Never drinker	1,652 (22.5%)	155 (16.0%)	
Abstention	2,253 (30.7%)	300 (31.0%)	
Current	3,424 (46.7%)	513 (53.0%)	
Aerobic exercise frequency (day/week)			0.07
Not at all	1,396 (17.3%)	151 (14.4%)	
<3days/week	1,410 (17.5%)	200 (19.0%)	
≥3days/week	5,247 (65.2%)	701 (66.6%)	
Strength exercise frequency (day/week)			<0.001
Not at all	5,870 (72.9%)	675 (64.2%)	
<3days/week	1,063 (13.2%)	178 (16.9%)	
≥3days/week	1,120 (13.9%)	199 (18.9%)	
Sleep duration			0.001
<5 hour	1,055 (13.1%)	186 (17.7%)	
5–9 hour	6,412 (79.6%)	793 (75.4%)	
>9 hour	586 (7.3%)	73 (6.9%)	

### Characteristics of shift workers with and without sarcopenia

There were statistically significant differences in energy intake, smoking status, strength exercise frequency, BMI, total fat mass index, HbA1C, HDL, and homeostatic model assessment for insulin resistance (HOMA-IR) index between shift workers with and without sarcopenia (Table 3).

**Table 3:** Comparisons between shift work with sarcopenia vs shift work without sarcopenia.

	Shift work without sarcopenia (N = 549)	Shift work with sarcopenia (N = 503)	*P*
**Age, mean age (SD)**	44.4 ± 14.4	46.7 ± 14.4	0.01
**Male n(%_)**	260 (47.4%)	455 (90.5%)	<0.001
Energy (kcal/day)	2,028.2 ± 921.5	2,373.8 ± 1074.8	<0.001
Excess	233 (49.5%)	296 (69.6%)	
Carbohydrate (g/day)	322.3 ± 131.7	369.4 ± 140.2	0.6
>65% of total energy	357 (35.0%)	168 (33.4%)	
Protein (g/day)	71.7 ± 39.0	86.7 ± 57.3	0.3
≥20% of total energy	119 (21.7%)	122 (24.3%)	
Fat (g/day)	41.0 ± 29.2	49.0 ± 43.8	0.7
>30% of total energy	113 (20.6%)	109 (21.7%)	
**Current smoking**	74 (13.5%)	102 (20.3%)	<0.001
**Current drinking**	182 (35.3%)	118 (26.0%)	
Aerobic exercise frequency (day/week)			0.2
Not at all	83 (15.1%)	68 (13.5%)	
<3days/week	110 (20.0%)	90 (17.9%)	
≥3days/week	356 (64.8%)	345 (68.6%)	
Strength exercise frequency (day/week)			<0.001
Not at all	380 (69.2%)	295 (58.6%)	
<3days/week	95 (17.3%)	83 (16.5%)	
≥3days/week	74 (13.5%)	125 (24.9%)	
Sleep duration			0.2
<5 hour	108 (19.7%)	78 (15.5%)	
5–9 hour	402 (73.2%)	391 (77.7%)	
>9 hour	39 (7.1%)	34 (6.8%)	
BMI	24.6 ± 3.6	23.2 ± 2.8	<0.001
>25			
Waist circumference (cm)			<0.001
low	368 (67.6%)	401 (80.7%)	
high	176 (32.4%)	96 (19.3%)	
Total body Fat mass group			<0.001
low	322 (58.7%)	367 (73.0%)	
high	227 (41.3%)	136 (27.0%)	
Laboratory data			
HbA1C, %	5.9 ± 1.0	6.6 ± 1.9	<0.001
Total cholesterol, mg/dL	189.2 ± 33.5	185.1 ± 37.3	0.07
HDL, mg/dL	49.8 ± 11.7	47.2 ± 11.3	<0.001
HOMA-IR*	3.2 ± 2.6	1.9 ± 0.6	<0.001

Data are presented as the mean ± SE, or percentage (SE).

Abbreviation: HOMA-IR, Homeostatic Model Assessment for Insulin.

### Odds ratio of sarcopenia according to shift work type in univariate and multivariate analyses

Sarcopenia was defined using different calculations, and all types of sarcopenia were associated with the type of shift work after adjustment for socioeconomic factors, health behaviors, fat mass, and laboratory indices (Table 4).

**Table 4:** Univariate and Multivariate analyses producing odds ratio of risk factors for sarcopenia among all types of workers.

	Sarocopenia	Sarcopenia_ht (N = 1,037, 11.4%)			Sarcopenia_wt (N = 1,802, 19.7%)			Sarcopenia_BMI (N = 3,377, 37.1%)		
	Variables	OR	95%CI	P	OR	95%CI	P	OR	95%CI	P
Shift work +	Crude OR	1.3	(1.1–1.7)	0.009	1.2 (1.1–1.5)	(1.1–1.5)	0.01	1.7	(1.5–1.9)	0.005
	Model 1	1.3	(1.1–1.7)	0.009	1.2 (1.0–1.4)	(1.0–1.4)	0.03	1.3	(1.1–1.4)	0.003
	Model 2	1.3	(1.0–1.7)	0.02	1.3 (1.0–1.6)	(1.0–1.6)	0.02	1.3	(1.1–1.5)	0.001
	Model 3	1.3	(1.0–1.6)	0.07	1.3 (1.0–1.6)	(1.0–1.6)	0.03	1.3	(1.1–1.6)	0.001
	Model 4	1.2	(0.9–1.6)	0.1	1.3 (1.0–1.6)	(1.0–1.6)	0.03	1.3	(1.1–1.6)	0.001
	Model 5	1.2	(0.9–1.6)	0.1	1.2 (1.0–1.5)	(1.0–1.5)	0.09	1.3	(1.1–1.5)	0.002

Model 1: adjusted for shift work type, age, sex,

Model 2: adjusted for shift work type, age, sex, BMI, fat mass, waist circumference.

Model 3: adjusted for shift work type, age, sex, BMI, fat mass, waist circumference, smoking, drinking, protein intake, carbohydrate intake, and fat intake.

Model 4: adjusted for shift work type, age, sex, BMI, fat mass, waist circumference,fasting blood glucose, metabolic syndrome, smoking, drinking, protein intake, carbohydrate intake, and fat intake, anaerobic exercise, aerobic exercise, and sleep duration.

Model 5: adjusted for shift work type, age, sex, BMI, fat mass, waist circumference, fasting blood glucose, metabolic syndrome, smoking, drinking, energy excess, protein intake, carbohydrate intake, and fat intake, anaerobic exercise, aerobic exercise, sleep duration, education, income, and depressive symptom.

## Discussion

In this nationally representative cohort study, we found that sarcopenia was more prevalent in shift workers than in those who had never experienced shift work. Shift workers had an increased risk of sarcopenia even after adjustment for potential confounding factors, including age, sex, low-protein diet, leisure time, physical inactivity, sleep duration, smoking, alcohol intake, insulin resistance, mental illness, and socioeconomic factors. Furthermore, among shift workers, irregularly scheduled shift work was more strongly associated with sarcopenia than regularly scheduled shift work. The results of this study suggest that the circadian rhythm disturbance associated with shift work may contribute to an increased risk of sarcopenia.

To our knowledge, this is the first study to reveal an association between shift work, which is associated with circadian rhythm disturbance, and sarcopenia in a large nationally representative population. Although previous studies have shown that sleep duration is related to sarcopenia, the effect of circadian rhythm disturbance on an increased risk of sarcopenia has not been studied in human populations, although it has been examined on an endocrinological basis in molecular studies^24–26^.

Interestingly, irregularly scheduled shift work was significantly associated with a higher prevalence of sarcopenia compared to regularly scheduled work, even after adjusting for age, sex, diet, physical activity, alcohol intake, and smoking. No previous studies have directly compared the prevalence of sarcopenia between subjects engaged in regular work and those with irregular shift work. Several preliminary reports indicated a higher prevalence of metabolic syndrome among irregularly scheduled shift workers than among regular shift workers. Sun *et al*. reported that irregular and unscheduled shift workers had higher rates of obesity than did those engaged in regular rotating night shift work^27^. Further large-scale prospective studies are needed to clarify this issue.

Several pathophysiological mechanisms may explain the relationship between shift work and increased risk of sarcopenia^24^. There is emerging evidence of crosstalk between circadian cycling and skeletal muscle metabolism^24^. A preliminary study suggested that the molecular clock, *MyoD*, and metabolic factors represent a potential system of feedback loops^28,29^. Beta-adrenergic signaling is considered a pathway involved in coordinating communication between circadian clocks in skeletal muscle^24^. Given that circadian rhythm is associated with skeletal muscle recovery and build-up and that shift work is associated with disruption of the circadian rhythm^21,27,30^, shift work and skeletal muscle mass index could be closely associated. However, to our knowledge, no human population-based studies have addressed this issue.

Disruption of the circadian rhythm is associated with the development of a variety of diseases in humans, including metabolic syndrome, cardiovascular disorders, and malignancy^6,7,9,15,31^. Circadian rhythm disruption may lead to disturbance of internal homeostasis for physiological processes and result in the development of pathologies^29^. Indeed, shift workers show high prevalence rates of the diseases outlined above^32^. Moreover, sarcopenic status is known to be closely associated with metabolic syndrome and its complications. The results of the present study indicated that shift work, which disrupts the circadian rhythm, may increase the risk of sarcopenia.

There were several reports that sleep duration <6 hours or >8 hours tended to be associated with sarcopenia. Kwon *et al*. reported that long sleep duration (≥9 hours) was associated with an increased risk of sarcopenia compared to 7 hours of sleep^19^. Lucassen *et al*. reported that poor sleep quality and later sleep times were risk factors for osteopenia and sarcopenia^20^. They reported that sleep parameters, including decreased sleep quality and later sleep times, were associated with increased risk of sarcopenia^20^. Kim *et al*. reported that sleep duration is closely associated with body composition measures^26^. They revealed that more than 9 hours of sleep duration is independently associated with sarcopenia, especially in women aged > 40 years. In our study, even though <5 hours or > 9 hours of sleep duration were more prevalent in the sarcopenia group compared with the non-sarcopenia group among shift workers, the relationship between sleep duration and sarcopenic status was not statistically significant.

Our study results showed that circadian rhythm disruption is independently associated with sarcopenia even after adjusting for sleep duration. In the present study, we adjusted for sleep duration to examine the association between shift work and the risk of sarcopenia, and we found that circadian rhythm disruption was independently associated with an increased risk of sarcopenia even after adjusting for sleep duration. Sleep duration and circadian rhythm disruption may be associated with sarcopenia in different manners.

This study has several limitations. First, although our results indicated a strong relationship between shift work and sarcopenia, this study should be interpreted cautiously because of its cross-sectional nature. Further large-scale population-based prospective studies are therefore required. Second, although we assessed sleep quality with an extensive questionnaire, other factors may be associated with sleep hygiene, such as obstructive sleep apnea. Obstructive sleep apnea is known to be a determinant of sleep quality and circadian rhythm disruption. Despite these limitations, this study had a number of strengths in that sarcopenia was assessed by dual-energy X-ray absorptiometry in a large nationally representative study population, and we measured extensive potentially confounding factors at baseline, including dietary patterns, leisure time, and physical activity, which are major determinants of sarcopenia.

In conclusion, we evaluated the association between shift work, which is known to disrupt circadian rhythm, and the risk of sarcopenia. As sarcopenia is a well-known risk factor for a variety of diseases, careful examination of sarcopenic status is required among shift workers and other groups with circadian rhythm disruption. Further prospective studies are needed to clarify the causal nature of the relationship between shift work and sarcopenia.

## Data Availability

The data used to support the findings of this study are available from the corresponding author upon request.

## References

[1] Beaudart C (2016). Sarcopenia in daily practice: assessment and management. BMC Geriatr.

[2] Santilli V (2014). Clinical definition of sarcopenia. Clinical Cases in Mineral and Bone. Metabolism.

[3] Sanches IC (2018). Combined aerobic and resistance exercise training attenuates cardiac dysfunctions in a model of diabetes and menopause. PLoS ONE.

[4] Lee SJ, Park YJ, Cartmell KB (2018). Sarcopenia in cancer survivors is associated with increased cardiovascular disease risk. Support Care Cancer.

[5] Shiraishi A (2018). Prevalence of stroke-related sarcopenia and its association with poor oral status in post-acute stroke patients: Implications for oral sarcopenia. Clin Nutr.

[6] Han, E. *et al*. Sarcopenia is associated with the risk of significant liver fibrosis in metabolically unhealthy subjects with chronic hepatitis B. *Aliment Pharmacol Ther* (2018). 10.1111/apt.14843 29920701

[7] Koo BK (2017). Sarcopenia is an independent risk factor for non-alcoholic steatohepatitis and significant fibrosis. J Hepatol.

[8] Lee YH (2015). Sarcopaenia is associated with NAFLD independently of obesity and insulin resistance: Nationwide surveys (KNHANES 2008–2011). J Hepatol.

[9] Jones SE (2015). Sarcopenia in COPD: prevalence, clinical correlates and response to pulmonary rehabilitation. Thorax.

[10] Polinder-Bos HA (2018). Lower body mass index and mortality in older adults starting dialysis. Scientific Reports.

[11] Kim JK (2014). Prevalence of and factors associated with sarcopenia in elderly patients with end-stage renal disease. Clin Nutr.

[12] Argeny S (2018). Visceral fat area measured with computed tomography does not predict postoperative course in Crohn´s disease patients. PLoS ONE.

[13] Schink K (2018). Effects of whole-body electromyostimulation combined with individualized nutritional support on body composition in patients with advanced cancer: a controlled pilot trial. BMC Cancer.

[14] Schreiner AJ (2018). Periprosthetic tibial fractures in total knee arthroplasty – an outcome analysis of a challenging and underreported surgical issue. BMC Musculoskeletal Disorders.

[15] Park YS (2017). Sarcopenia is associated with an increased risk of advanced colorectal neoplasia. Int J Colorectal Dis.

[16] Heo JE (2018). Association between appendicular skeletal muscle mass and depressive symptoms: Review of the cardiovascular and metabolic diseases etiology research center cohort. J Affect Disord.

[17] Perkisas, S. *et al*. Prevalence of sarcopenia and 9-year mortality in nursing home residents. *Aging Clin Exp Res* (2018). 10.1007/s40520-018-1038-2 30218406

[18] Matsumoto T (2018). Associations of obstructive sleep apnea with truncal skeletal muscle mass and density. Sci Rep.

[19] Kwon YJ (2017). Long Sleep Duration is Associated With Sarcopenia in Korean Adults Based on Data from the 2008–2011 KNHANES. J Clin Sleep Med.

[20] Lucassen EA (2017). Poor sleep quality and later sleep timing are risk factors for osteopenia and sarcopenia in middle-aged men and women: The NEO study. PLoS ONE.

[21] Boivin DB, Boudreau P (2014). Impacts of shift work on sleep and circadian rhythms. Pathologie Biologie.

[22] Kweon S (2014). Data resource profile: the Korea National Health and Nutrition Examination Survey (KNHANES). Int J Epidemiol.

[23] Safiri S, Ayubi E (2017). Sarcopenia based on the Asian working Group for Sarcopenia criteria and all-cause mortality risk in older Japanese adults: Methodological issues. Geriatr Gerontol Int.

[24] Pearen MA (2009). Expression profiling of skeletal muscle following acute and chronic beta2-adrenergic stimulation: implications for hypertrophy, metabolism and circadian rhythm. BMC Genomics.

[25] Lynch GS, Ryall JG (2008). Role of beta-adrenoceptor signaling in skeletal muscle: implications for muscle wasting and disease. Physiol Rev.

[26] Kim RH (2018). Association between Sleep Duration and Body Composition Measures in Korean Adults: The Korea National Health and Nutrition Examination Survey 2010. Korean Journal of Family Medicine.

[27] Sun M (2018). Night shift work exposure profile and obesity: Baseline results from a Chinese night shift worker cohort. PLoS One.

[28] Harfmann BD, Schroder EA, Esser KA (2015). Circadian rhythms, the molecular clock, and skeletal muscle. J Biol Rhythms.

[29] Lefta M, Wolff G, Esser KA (2011). Circadian rhythms, the molecular clock, and skeletal muscle. Curr Top Dev Biol.

[30] Marqueze EC, Ulhoa MA, Moreno CR (2013). Effects of irregular-shift work and physical activity on cardiovascular risk factors in truck drivers. Rev Saude Publica.

[31] Jeong HG (2017). Physical Activity Frequency and the Risk of Stroke: A Nationwide Cohort Study in Korea. Journal of the American Heart Association: Cardiovascular and Cerebrovascular Disease.

[32] Chaudhari A (2017). Circadian clocks, diets and aging. Nutr Healthy. Aging.

